# ‘It is important for us to see the mentors as persons’ – participant experiences of a rehabilitation group

**DOI:** 10.1080/17482631.2019.1632108

**Published:** 2019-06-26

**Authors:** Josephine Andreasen, Stig Poulsen, Michaela Hoej, Sidse Arnfred

**Affiliations:** a Department of Psychology, Faculty of Social Sciences, University of Copenhagen, Copenhagen, Denmark; b Department of Public Health, Faculty of Health and Medical Sciences, University of Copenhagen, Copenhagen, Denmark; c Competence Centre for Rehabilitation & Recovery, Mental Health Centre Ballerup, Ballerup, Denmark; d Psychiatry Vest, Psychiatric Hospital Slagelse, Slagelse, Denmark

**Keywords:** Recovery, therapeutic alliance, educational support, youth, mentoring, coaching, community intervention, group therapy, authenticity, empowerment

## Abstract

**Purpose**: The purpose of the study was to examine what was beneficial and what was challenging in a group intervention for young adults based on RENEW principles in a municipal employment centre. RENEW (Rehabilitation for Empowerment, Natural support, Education, and Work) is an education-oriented support model for young people.

**Method**: The eight young adults who participated in the group and three mentors who led the group were interviewed about their experiences with the group, and a workshop was held for staff to validate the themes found in the study.

**Results**: Three themes emerged, one denoted the importance of *helpful personal relationships*, both between the mentors and the young adults and among the young adults; another denoted how an authentic attitude from the mentors made *group exercises inconspicuous* as the group members experienced activities in the group as originating from spontaneous, genuine interest rather than the manual-based exercises they were. The last theme conveyed how the group process was challenged by the institutionally regulated *compulsory attendance and the mentors’ lack of teamwork resources*.

**Conclusions**: The study suggests that meeting young adults authentically and flexibly combining a certain element of self-disclosure with a manual-based group intervention such as RENEW can strengthen relatedness and convey hope, thereby supporting educational rehabilitation.

## Introduction

While recovery-oriented approaches are increasingly the main path for supportive community interventions in mental health services (Amering & Schmolke, 2009; Davidson, O’Connell, Tondora, Staeheli, & Evans, 2005; Shepherd, Boardman, & Slade, 2008; Slade, 2010), they are less prominent in municipal back-to-work or -education interventions. However, a recovery-oriented approach also seems relevant for support and prevention among vulnerable young people without known psychiatric diagnoses. The emphasis on the users’ feelings of control, empowerment, hope, the focus on strengths and future, and the feeling of being connected to others, to one’s social network or community (Ness, Borg, & Davidson, 2014; Schön, Denhov, & Topor, 2009; Tew et al., 2012) are highly relevant for this vulnerable group. One programme which utilises the recovery-oriented approach is the education-oriented support model RENEW (Rehabilitation, for Empowerment, Natural support, Education, and Work). RENEW was developed by a group of American researchers and practitioners in 1996 as a system for supporting young adults (14–21 years) with emotional and behavioural problems who were in risk of, or in fact, dropping out of the education system (Cheney, Hagner, Malloy, Cormier, & Bernstein, 1998; Malloy, Drake, Cloutier, & Couture, 2012). The purpose of the model is to promote the young adults’ social skills, to develop self-determination, activate the young adults’ network, plan the transition from school to work life and support the young adults with a strength-based perspective. The original RENEW model was based solely on individual sessions and network meetings, but we have developed a group module, manualized in close correspondence with the principles of the individual sessions (Hoej & Arnfred, 2015). Having added this new group intervention to RENEW, we wish to explore which elements the users experience as beneficial, and which they experience as disadvantageous in the new group intervention in a municipal setting.

Numerous studies show that group therapy is effective for adolescents and young adults, e.g., adolescents who are deliberately self-harming (Wood, Trainor, Rothwell, Moore, & Harrington, 2001), young adults with social phobia (Piet, Hougaard, Hecksher, & Rosenberg, 2010), and young adults with personality disorders or personality disorder features (Renner et al., 2013). Knowledge is, however, lacking as to which therapeutic factors and processes are important or inhibitory in group therapy for adolescents and young adults, as well as why group therapy can be especially helpful for young people (Kymissis, 2007; Oetzel & Scherer, 2003). The present study aims to contribute with new perspectives on beneficial active ingredients of groups for young adults.

Not many studies include both consumer and provider perspectives in research though some research shows that the perceptions about problems (Klinkenberg, Cho, & Vieweg, 1998), needs and services (Crane-Ross, Roth, & Lauber, 2000), and outcome can differ considerably (Crane-Ross, Lutz, & Roth, 2006; Eisen, Dill, & Grob, 1994; Murnen, 2002). For example, Crane-Ross et al. (2006) found that consumers’ and case managers’ perceptions about service empowerment were related but the level of agreement was relatively low. The authors argue that the discrepancy may in part be due to the consumers’ and case managers’ different views about the types of activities or relationships that contribute to empowerment. They state that for example, the case managers may view the work with the consumers on an individual service plan as an empowering activity, while consumers may be influenced by more subtle aspects of their relationship with the case manager and may not necessarily perceive this activity as empowering in and of itself (Crane-Ross et al., 2006). Studies reporting experiences of providers of care in mental health outpatient services underline the difficulties associated with delivering recovery-oriented care, where provider ambivalence and organizational-systemic barriers (Piat & Lal, 2012) as well as the difficult layers of negotiation with the users (Kvig, Moe, Brinchmann, Larsen, & Sørgaard, 2019) are experienced as challenges. Furthermore, mental health professionals working with adolescents describe an additional challenge being the difficulty of engaging families in a relevant manner and thereby perhaps resulting in working with crisis management at the cost of futures planning (Grube & Mendenhall, 2016). Social workers supporting youth in schools also describe lack of resources, while they see the relational and emotional support as making the most important difference for the young person (Anderson, 2017). These findings do not pertain particularly to group interventions, but they suggest that it is important to include perspectives of both consumers and providers when conducting research.

We therefore examine how the participants, i.e., the young adults and group mentors, experience the RENEW group sessions, with a special focus on what the participants experience as beneficial and disadvantageous in the group, and conducive to a positive outcome.

## Methods

### Ethics

The study was conducted in accordance with the Code of Ethics of the American Anthropological Association and the research conforms to the ethical principles for medical research on human beings set out in the declaration of Helsinki). The study was approved by The Institutional Ethical Review Board, University of Copenhagen, Department of Psychology (IP-IRB/15052019).

Informed consent was obtained from all the participants at the interviews, and the participants received both verbal and written information about the project and that they could withdraw from the project at any time without any explanation. The data was anonymised and processed and stored in accordance with the Danish Data Protection Agency regulations. Thus, it is not the participants’ real names, which appear in the article.

### Setting

In Denmark, people who receive aid from public welfare services are obliged by law to attend meetings in municipal employment centres, where job specialists provide assistance in finding employment or education. If people fail to attend these meetings, their social welfare services are reduced. The current study took place at a community education centre (CEC) from December 2016 to July 2017. CEC’s purpose is to help unemployed and uneducated young adults aged 18–30 to prepare themselves for or complete an education through mentoring, guidance, and teaching. Many of the young adults have a psychiatric vulnerability. Prior to this study, the young people had been able to register absences from the youth group through their mentor, but at the time of the research project it had just been made compulsory to participate in group sessions. Hence, young adults were to call a municipal official, should they need to call in sick. If they did not report being either sick or late, the mentors were obliged to register their absence, which could potentially lead to a reduction in their social support payments.

### Participants

To recruit the participants, an email with information about the interviews was send to the mentors, who then communicated this information to the young adults. Once the young adults and the mentors had agreed to take part in the interview, they received an email with relevant practical information.

#### Young adults

Eight young adults between the ages of 23 and 29 were interviewed. The eight participants were selected in dialogue with the mentors with the aim of having a representative sample of present and former members of the group with regard to gender, age and number of group sessions they had experienced. Two of the eight participants were no longer in the group. Four of the participants had been in the group long enough to have been through the eight themes covered by the group sessions several times. The young adults will be presented as: Laura, Lotte, Ditte, Benjamin, William, Thea, Nanna and Dennis.

#### Mentors

All mentors who worked with the group were selected for the interview. Two of the three mentors interviewed had worked with RENEW since the start of the project and had taken part in a workshop in December 2014 where the American developers of RENEW taught them how to work with the RENEW model. The last mentor was hired for the RENEW project six months later and also received training in the RENEW method. Their respective educational backgrounds were: nurse, nurse’s aide and master’s degree in social sciences. The three mentors will be presented as: S, J and C.

### Intervention

RENEW comprises procedure-based individual coaching or mentor support as well as help in making use of and building up support networks around the young people. After the initial positive results (Bullis & Cheney, 1999; Malloy & Cormier, 2004), a small descriptive study with 20 teenagers showed promising effects after a 1-year intervention on mood, self-harm and daily function level (Malloy, Sundar, Hagner, Pierias, & Viet, 2010).

RENEW focuses on five principles, related to the concepts of recovery, which can be seen in Table I (Malloy et al., 2012, 2010, p. 1) Self-determination implies that the young adults obtain skills in problem solving, choice making, self-knowledge and help seeking. This principle builds upon Ryan and Deci (2008) self-determination theory, which presents three universal psychological needs: competence, autonomy and relatedness. 2) The principle of community inclusion focuses on supporting the young adults in taking an active part in their local community, and in using the resources there, e.g., their social relations. 3) The principle of unconditional care comprises an understanding that the young adults cannot lose their support or be excluded from their RENEW treatment if they are not compliant 4) The principle of strength-based planning implies that the work with the young adults must focus on their strengths rather than their vulnerabilities and on their abilities to plan their future and goals. 5) The final principle of flexible resources is based on an understanding that support for the young adults must be tailored to the young people’s needs and not designed according to the types of support which are available.

**Table I.** The five principles of RENEW.

The Five principles of RENEW
1. Self-determination
2. Community inclusion and authentic support
3. Unconditional care
4. Strengths-based planning
5. Flexible resources

The RENEW model prescribes different phases for individual work with the young adults where the first phase is a graphic facilitation phase. In this phase a mentor draws up a specific action plan for the young adults in partnership with the young people themselves. Here, the RENEW mentor draws and writes on flip-charts based on what the young adults say. Themes in this phase are 1) *my story*, 2) *who you are today*, 3) *strengths and things I have succeeded in*, 4) *my network*, 5) *what works—what doesn’t work*, 6) *my dreams, concerns, barriers and challenges*, 7) *my goals* and 8) *the next step*. A new flip-chart is drawn for each theme. The final flip-chart (the next step) is the action plan for the young adults’ next step towards achieving their goals.

The next phase deals with working with the action plan. As far as possible, the young adults carry out the work themselves with support from their network and the RENEW mentor. The idea is to take advantage of the resources that exist in the young adult’s own network; both professional and personal connections, who can facilitate contacts or offer support. Throughout this process, the RENEW mentor works to increase the young adults’ coping skills. This is achieved by supporting them in trying to solve challenges themselves. In the final phase, the mentors help the young adults to manage the future without professional help, while continuing to receive support from their personal network.

#### The RENEW group

The group was not originally part of the RENEW model, but due to an expressed desire from both the mental health and the employment centre, a group intervention was developed in collaboration with the mentors as a supplement to RENEW in Denmark (Hoej & Arnfred, 2015).

In the group, the RENEW mentors take turns in leading it in teams of two mentors once a week. The group is conducted in an open format; hence young adults stop and start on an ongoing basis, thus there is no specific date for when they will stop being in the group. The eight themes that form the structure of the group are based on the themes that are used in the graphic facilitation phase in the individual work. These eight themes run in a loop, and each of them offers several suggestions for exercises which the mentors can choose from, with two exercises selected for each group session. A group session lasts two hours and generally follows the plan shown in Table II. However, in some sessions, there was insufficient time to fit in all the exercises or discuss which questions the young people wanted to work on for next time.

**Table II.** RENEW general group session agenda.

RENEW GROUP SESSION AGENDA
- Check-in exercise
Pick a card or several, which for you shows a picture of what you are concerned about, or how you feel at this particular moment
- Have you worked on anything from the last session?
- Activity or Exercise (Topic according to manual)
− 10 min. break
- Activity or Exercise (Topic according to manual)
- What will you work on for next time?

An example of a group exercise is where one young adult presents his or her text on a specific flip-chart, e.g., ‘Strengths and things I have succeeded in’. The purpose of presenting their own flip-charts is to train them in talking about themselves, thereby becoming better at informing their network about how they are doing. In addition, participants in the group can also exchange experiences and support each other based on the presentation. To obtain an understanding of the practice the first author was an observer in the group before conducting the interviews.

### Data collection

Data consisted of group interviews, initially with young adults, subsequently with RENEW group mentors and finally in a workshop with the mentors, where the mentors validated the data from their own interview.

### Interviews

The young adults were interviewed in three groups; three young adults in one interview group, three in another, and two in the last. The three mentors leading the group were interviewed in a group interview. In this interview, the manager and a second RENEW mentor were present as witnesses, which meant that they listened to the mentor’s experiences and at the end of the interview shared how it resonated to them. The practice of outsider witnesses originates from narrative therapy and is frequently applied in narrative interviews with the intent of enchancing the feeling of community and support between the participants (Fredslund, 2013; White, 2008). This aim of the practice of outsider witnesses in narrative interviews is to strengthening the ongoing work between co-workers and was therefore only used in the interviews with the mentors. All the group interviews were conducted primarily as individual interviews in the group, but the other group members were allowed to contribute their thoughts and opinions during the individual interviews (Flick, 2009). The young adults and the mentors were asked in an email sent out prior to the interview to think of beneficial (worked well) and disadvantageous (was difficult) experiences what was in the group to give them an opportunity to come up with specific experiences from the group. Each interview began with a young adult or mentor presenting one of their prepared examples.

The interview guides were inspired by Fredslund’s narrative interview method (Fredslund, 2013; Nielsen, Fredslund, Christensen, & Albertsen, 2006). The interview guide was broken down into themes, which acted as markers, in accordance with Fredslund’s (2013) recommendations and in line with Michael White’s (2008) method of navigation in therapeutic interviews. Hence, all the themes were covered, without necessary having to ask the complete set of questions for each theme. The interviews can thus be classified as semi-structured interviews, with the necessary flexibility to ensure elaboration of the responses (Kvale, 2007). All the interviews were conducted by the first author and took place at CEC.

The duration of the three interviews with the young adults varied from between one hour and 56 minutes and two hours and 11 minutes, while the interview with the mentors lasted for two hours and 45 minutes. All statements from the interviews included in the present article were translated by a professional translator and the translation was subsequently compared with the original interviews to make sure the meaning was maintained.

### Workshop

After the interviews, a workshop was held where the themes from the young peoples’ and the mentors’ interviews were presented while the mentors and the manager were present. The workshop had two purposes. First, it allowed the mentors to validate and elaborate on the themes of their interview. This additional material was integrated into the final version of the themes of the analysis. Second, the workshop gave the mentors the opportunity to discuss how the results could be used in their continuing work, focusing on the young people’s suggestions to how the mentors could strengthen or alter practices in the group. The workshop lasted for two hours and 29 minutes.

### Data analysis

The data analysis was conducted by the main author. No computer programs were used for the data analysis. First, an inductive condensation of meaning was performed to find a theory-neutral presentation of the participants’ experiences (Ekroll & Rønnestad, 2016; Hsieh & Shannon, 2005). After this, a theoretically-focused discussion was carried out (Hsieh & Shannon, 2005). The condensation of meaning was done in four steps.

Initially, the interviews were read one at a time, first in their entirety and secondly with emphasis on statements that were relevant to the research question. Subsequently, keywords were added in the margins next to underlined statements, followed by a rewrite of the statement into brief units of text aiming to express the meaning of the statement (see Table III for an example of the coding process). The interviews were then read again with a focus on organizing the statements into themes of what worked well and what could be difficult in the young adult group. The themes were combined, separated and their names changed in an iterative process, and sub-themes were created. The researcher paid attention not only to statements that represented experiences shared by all the participants, but also to differences in the participants’ experiences of what was beneficial and what was disadvantageous in the group.

**Table III.** Example of the data analysis process for the theme *organisational challenges: compulsory attendance and lack of teamwork resources.*

Statement	Keywords	Description and understanding
*“ … there is this contrast, that here in the group they are considerate and understanding, but the bureaucracy and the reception and all that, it’s more strict, I think. It’s very much that with being off sick and being punished for being off sick and the suspicion, they have of you … ”*	Sense of security, consideration and understanding in the group. Contrast to reception. Punishment for being absent. Suspicion.	Ditte’s experience is that the processes in the young adult group are in great contrast to what she experiences when she registers as sick. While the group is equated with a sense of security, consideration and understanding, the method of registering as sick is equated with bureaucracy—which is described as strict, punishing and distrustful.
*“She is also more of a person than him down in reception. I don’t think that it would matter if I was to report all my sickness absences to her and then write her a note as to why I had not been there today.”*	Humanity. Registering sickness with the mentors.	Nanna describes the mentors as being more like people (sympathetic) than the people down in the reception.
*“ … Perhaps I needed to do something good for myself, but it ended up with me spending an entire day just lying under the duvet at home because it was really all I could manage. I’m the kind of person who feels guilty and … I couldn’t even allow myself to sit out in the sun, because then I would not really be sick. And just sitting out in the sun; that might have helped me to feel a bit better the next day.”*	Distrust. Feelings of guilt. This cannot be right. Not really sick. Limiting. A possible obstacle to recovery.	Nanna talks about an experience where she was met with suspicion when she registered as sick. Her reaction was that this could not be right, but this was also accompanied by feelings of guilt and thoughts that perhaps she wasn’t really sick, and therefore she could not allow herself to sit out in the sun, for example, which might have helped her to feel better.
*“For me it all triggers that social stigma … And then it will ruin my entire day, and then I’ll think that I’m just lazy and not really sick. Mental illness is apparently not a real illness and then you get that … guilty conscience and just feel completely in the wrong.”*	Social stigma. Laziness. Burden on society. Mental illness is not a real illness. Limiting. A possible obstacle to recovery.	Ditte describes how registering as sick can trigger a social stigma, which triggers a view of herself as lazy and not really sick. She says that it can ruin her entire day, and that she will have a bad conscience and feel like she has done something wrong. The young people describe otherwise how the group has helped them to not feel in the wrong.

Following the principle of respondent validation (Silverman, 2015), the inventory of themes and sub-themes was presented at the workshop with the mentors. The mentors approved their own statements from the interview and discussed how they could understand and use the statements from the interview with the group members in the daily work with the group. The authors subsequently discussed the categories and sub-themes, and the final three themes were determined based on a mutual understanding of the results.

## Results

In the following, the three themes will be presented. In the category *Helpful personal relations*, the young adults highlighted several relational aspects of the group. They spoke about the importance of being able to share experiences with each other in the group and about the significance of their experience of the mentors as authentic and caring. The mentors also expressed that they tried to behave authentically when carrying out exercises with the young people in the group. The theme, *Inconspicuous Group Activities*, focuses on how the young people did not necessarily experience the exercises as pre-planned structured activities but rather as spontaneous activities initiated by the mentors out of pure interest. In the theme, *Compulsory Attendance and Lack of Teamwork Resources*, the young people and the mentors’ experiences of the negative consequences of the institutional framework for the young adult group are in focus. They described how the institutional framework can have negative consequences for the young people’s rehabilitation, limit the mentors in their work and affect the personal relationship between the young adults and the mentors, which, as previously described, was one of the things the young people especially valued.

### Helpful personal relations

All the young adults stated that their relationship with the mentors was important. Thea said that she was especially pleased when the mentors involved themselves personally by talking about their own lives: *“Then they are not just adults, who sit and keep an eye on us, but more like … they are part of the group.”* The positive thing, according to Thea, was that *“[…] Then you start to see them a bit like a friend. You don’t see them as someone you cannot trust […] It gives a sense of security, that they sit there and tell you something personal, then I can also sit down and talk about something personal.”* Laura added: “*It is important that we can see them [the mentors] as persons and not just as some guys who work for the municipality … It makes me feel more secure. Like I am being taken seriously.”*

William also believed that the reason why it was easier to participate in the group was that the mentors had an authenticity that seemed contagious: *“Every single time they open their mouth, it seems natural … And this also means that every single time we open our mouths it also seems natural.”* Besides being natural, Ditte and Lotte also felt that the mentors were not judgmental or focused on the performance of the group members, which for Ditte was one of the most important things about the group. She felt that the mentors’ intention of not being judgmental or performance-oriented helped to “*create a sense of security that we can open up here, I think. And that we want to be here. So, it is a positive thing.”*

Many of the young people noted that the mentors expressed that they were very committed to and happy to be involved with the group. For instance, Lotte said that: *‘you can also often hear them say things like … “this doesn’t feel like part of my job’, it is just the best, you can really feel something like … what can I say, love … ”*

Nanna also enjoyed the group because there was room for what she called ‘the personal’: *“ … It’s to do with us being persons, isn’t it? We’re not just a number. This is important in terms of gaining something from the group. It’s important in terms of us not just sitting and being crossed off on the register. That [being crossed off] isn’t something that can help you develop if you are having a hard time*.”

Laura said that it was important for her that there was room to go beyond the theme in the group: *“ … It could be that they have some kind of plan for the day, where we need to talk about goals for example, but there can also be room for, I think, if there’s someone who has something else on their mind which might go slightly beyond the day’s theme, then there is room for that as well […] this is nice and shows that they truly see the individual*.” Sometimes, the sharing of advice and experiences could take up so much time that they did not complete all the exercises. Laura found that this was a good thing because *“you also get some input from the others … and also perhaps some of the others, who are in the same situation as us and not our mentors.”* According to Benjamin, sharing their experiences presented an opportunity to hear about the others’ development, which could offer hope: *‘And then you think, you know, that if he can do it, then maybe I can too. There is some hope’*. This hope for the future, gained from the young adult group, highlighted Thea and Benjamin as being especially important.

Dennis, however, also saw a disadvantage in sharing advice in the young adult group: *“I would say that there is one small problem with all of this, and that is that it can quickly turn into a sort of ‘coffee club’ … ”* When the group resembled a ‘coffee club’, that could result in there being no time left to complete the exercises, or that the exercises had be rushed through, which Dennis found problematic.

Another relational element which worked well in the group, according to Dennis, was that they were *‘all in the same boat’*. According to Laura, the experience that everyone was in the same boat made her feel less alone and abandoned: *‘Then you get a sort of “Oh, I’ve tried that too, I’m familiar with it” […] you are more conscious of the fact that the people sitting with you actually have the same feelings and the same thoughts, and that you are not just sitting alone and abandoned’*. Lotte also described, that *“When I leave here, then I feel more ‘right’, you know? Or maybe not more right, but at least less wrong.”*

Many of the young people emphasised the fact that being in the same boat enabled them to use the group to practice improving their social skills. Thea said that she was able to “*use the group as a sort of playground*.” For her, the group was a place where she could develop her social skills, in particular she could practice openness and honesty in her relationships: *‘I haven’t really been able to open up, so having an opportunity to do this and practice doing it a lot […] made me feel better’*. Ditte added: *“I have gained more courage to be the centre of attention in other contexts out in the real world, I think.”* She said that this had to do with them all being equal: *“For me what’s almost most important is that there is this free space where we gain an understanding of each other, and it’s much easier here to open up and speak honestly. […] Here, we are pretty much all the same.”*

Lotte pointed out, however, that it could also be hard if young people were invited into the group who were not ready to be part of the community. In her experience it could create uncertainty if a person in the group did not appear to be ready to open up: “*It can perhaps create a kind of imbalance, which can be a little … bad for the group … I begin to feel a little like I’m sitting here taking all the attention and saying too much or … didn’t we do enough to make it feel comfortable for her to feel like saying something? Am I bad at making her feel welcome or have I overstepped her boundaries?”* Nanna agreed that it could affect the others in the group when someone did not say anything, because then *“It hinders all of us in the group; if you are sitting there thinking that it’s you who did something wrong.”*

This point was also addressed in the interview with the mentors. Thus, S agreed it could be difficult when some spoke more than others. It was important to S that the quieter young people could also have a positive experience by saying something if they wanted to do so. Furthermore, the mentors sometimes found it challenging to balance being controlling or non-controlling. C experienced this as especially challenging. On the one hand she wanted the young people to get something out of the exercises *‘so that they become more skilled at something and can progress with something’*. On the other hand, though, she realized that the young people ‘*really enjoy hanging out with each other*’ and may gain just as much from this as from the organized exercises.

Just like many of the young people, the mentors felt that the group was helpful in several ways. Thus, it both allowed the young adults to offer advice and share experiences with each other and gave the group members the opportunity to help each other in other ways, e.g., with practical things or by going to events together. J stated that one of her intentions was to facilitate the young people in helping each other, because this made them become: *“more self-reliant, so that they don’t only have to think that now we have to go down and see the lady at the municipality to get some help, but they also actually … with someone who they actually consider slightly as a friend and see sometimes in their free time, manage to solve some of the challenges which they have.”*

S also remarked that one of the effects of the young people helping each other was that those who have been helped are: *“being welcomed into the fold, so to speak. And this can give a feeling of success. That you can count on being seen.”* C agreed and mentioned an episode, where she noticed that those who helped also gained something from it: *“if you are able to give or offer something, then it is actually pretty cool, because many of them actually do not have very many resources.”*

### Inconspicuous group activities

As previously described, some of the young people said that it was important for them to have the opportunity to go beyond the day’s exercises. While Lotte and Dennis shared this opinion, they also (as the only young adults) stated that they found the exercises important in themselves. Lotte said: *“ … I think that the topics we work with each time, and the exercise that we do each time, gives me some kind of tool or a new way of looking at things. This approach of putting things into tables and boxes works extremely well for me.”*

Dennis, Benjamin and Lotte highlighted the importance of doing physical activities such as softball and football, or going to Open House meetings at educational institutions, visiting services for the psychologically vulnerable, or going to a museum. They perceived these activities as distinct from the usual exercises and believed that they could be featured more prominently in the group. Dennis felt that the reason these alternative activities, e.g., going on excursions, functioned well was that they could contribute to a sense of community in the group, which was important for making him feel interested in coming. Lotte pointed out that the different activities could help to get the young adults to trust one another. And Benjamin said that the different activities could provide the opportunity to visit some of the services available which could be hard to do alone, but which many of the young people wanted to see.

In the mentors’ interview, C, among others, remarked that: *‘[…] we notice that it is the exercises which makes them move forward’*. It therefore surprised the mentors that only two of the young people highlighted the exercises as being important. J said that they as mentors were skilled in making the exercises appear natural, which could mean that the young adults did not consider the questions asked in the group as being part of an exercise rather than just stemming from the mentors’ own personal interest. C recalled that a new check-in exercise could confirm what J said: “*[…] in reality we go a little deeper into some things than we have done previously and they get the opportunity to do a lot of talking … and while this is an exercise for us, perhaps it does not seem that way in their heads.”*

C also believed that the reason why the activities did not seem like programmed exercises could be that the young adults took ownership: *“I think it is because they asked for the paper themselves, so it becomes their personal whatever, so they don’t think of it as an exercise, because it’s different. And it came from somewhere else; it came from them.”*

### Compulsory attendance and lack of teamwork resources

The compulsory attendance requirement and the new method for calling in sick seemed to have a negative impact on the group members’ emotional and psychological gains from the group as well as their relationship with the mentors. In the interview with the mentors as well as particularly one of the interviews with the young people, this theme took up much of the interview, both in terms of time and intensity. It became very clear that both the mentors and in particular three young people, Nanna, Ditte and Lotte, experienced the compulsory attendance and method for calling in sick as being unnecessary and having negative consequences.

Nanna, Ditte and Lotte noted distrust and suspicion when they called in sick, and Nanna said: *“[…] I had the feeling that he [the receptionist] did not really believe me, and that it simply wasn’t true […] I’m the kind of person who feels guilty and … I couldn’t even allow myself to sit out in the sun, because then I wouldn’t really be sick. And just sitting out in the sun; that might have helped me to feel a bit better the next day.”* Thus, Nanna notes that this method of calling in sick could constitute a potential obstacle to her getting better.

While several of the young adults, as previously described, mentioned the realization that they are not “wrong” as one of the particular gains of the group, Ditte states that the experienced suspicion which she felt when calling in sick could facilitate a feeling of being in the wrong and rejected and ruin her day: *“For me it all triggers that social stigma […] And then it will ruin my entire day, and then I’ll think that I’m just lazy and not really sick. Mental illness is apparently not a real illness and then you get that … guilty conscience and just feel completely in the wrong.”* Ditte also described having valued the group as a place where there was no performance pressure, but when attendance became compulsory, she felt more of a pressure, affecting her negatively.

According to the mentor, J, the new method for reporting sick days also deprives the mentors of the opportunity to motivate the young adults to come and on following up on them if they should need it. And C described how the compulsory attendance of each session made it harder to build a positive relationship with the young adults: *“I think that it’s absurd to have to step into a set of values that deal with economics and a framework, and now I have to come after you with a whip, when I have also been asked […] that we should work on the relationship. I have no relationship whatsoever with people who carry whips in their hands.”* The other mentors acknowledged C’s descriptions.

The mentors also described other institutional challenges imposed on the group. J found it challenging that there was no continuity in the work with the group, both between each group session and between the individual RENEW programs and the young adult group. She wished that she could ‘*have had a greater overview and worked more with processes and the individual’s process’*. According to the mentors, continuity was lacking because there was no time to write down what the theme was for the group last time or what had happened, which made preparing for the next session difficult. The lack of time could also be a barrier in having mentor meetings, which further diminished the sense of continuity.

## Discussion

In the results, the young people’s relationship to the mentors it highlighted as extremely significant. The young adults valued the mentors being authentic, meeting them at their own level, and showing an interest in them as people. They also found it important that the mentors were not judgmental or focused on performance but rather were understanding and supportive. According to the young people, the mentors’ approach to them as equals was apparent in the way they shared personal details about their own education, working life and feelings. According to many of the young adults, this resulted in forming some kind of a friendship with the mentors; thereby gaining the courage to open up in the group.

According to the mentors, they also behaved natural when they carried out exercises in the group. Therefore, the exercises did not appear to be assignments described in a manual, but they were rather experienced by the young people as questions asked out of interest. With the emphasis the young people placed on their relationship to the mentors, it is not surprising that the compulsory attendance and duty to report all sick days was noted by both the young adults and the mentors as being especially problematic, because it made building up a relationship and close contact difficult.

### Authenticity in professional relationships

Humanistic psychology in particular has focused on authenticity in the therapeutic relationship (Moyes & Miller, 2013; Rogers, 1959; Schnellbacher & Leijssen, 2009). Authenticity, or *congruence*, is included in Carl Rogers’ ‘necessary and sufficient conditions of therapeutic personality change’ (Rogers, 1957) and a large number of subsequent studies suggest that the therapist’s congruence/authenticity may contribute positively to the success of the treatment (Kolden, Klein, Wang, & Austin, 2011). One phenomenon which is strongly linked to authenticity is self-disclosure, and many studies have shown that self-disclosure, if used correctly, can create a sense of security and help clients open up about themselves, which can contribute to the therapeutic alliance and affect the outcome of the therapy (Henretty, Berman, Currier, & Levitt, 2014). Precisely this use of self-disclosure was highlighted by the young adults as one of the things which they valued in the mentors and one of them described this as contributing to a close and friendly relationship. Many studies also show that, especially in group therapy with young adults, elements such as self-disclosure, authenticity and personal involvement can be important and contribute to facilitating a group climate where individuals can share their experiences with each other (Leader, 1991; Oetzel & Scherer, 2003). It must be noted that this kind of approach in therapy is not necessarily effective for all young people, as some find it too obtrusive, too direct, or strange and confusing (Greenberg, Watson, Elliott, & Bohart, 2001). This did not, however, appear to be the case for the young adults in this group.

The RENEW model emphasizes that it is essential that the mentor and the young adults is having a close relationship built on trust, and that it is the mentor’s task to support the young people throughout the process (Malloy, 2013; Malloy, Cheney, & Cormier, 1998). RENEW builds on principles from the recovery perspective as well as Ryan and Deci’s self-determination theory, both of which emphasise the importance of relationships. Ryan and Deci (2008) self-determination theory has, on the basis of theory and research, set out three universal psychological needs which are also valid within the therapeutic context: competence, autonomy and relatedness. The psychological need for relatedness is based on the premise that it is important to feel attached to other people and to feel that other people care about you. The therapist must therefore enter into a warm, genuine relationship with the client (Ryan & Deci, 2008). This is in accordance with the statements in the young people’s interviews.

Research in recovery also shows that having ‘relationship capital’ can be important in experiencing recovery (Tew et al., 2012). Relationship capital involves having significant others who can be affected by one’s experiences without being overwhelmed by them. In the interviews, the young adults highlighted this as helpful by noting that the mentors seemed genuinely interested in them.

The young adults described how, in the municipal regime, they were often met as cases to be handled, but they experienced that the mentors, in contrast to this, were interested in understanding and looking after them as people. Interestingly, several studies (Miller, Benefield, & Tonigan, 1993; Najavits & Weiss, 1994) indicate that it can be particularly meaningful for vulnerable people to be met with what Moyes and Miller (2013) call *accurate empathy*. The term is borrowed from Carl Rogers (1959, 1975)) and covers the therapist’s engagement in and ability to understand the client’s world and tune into the client’s needs and reference framework in the moment rather than analysing and assessing the client (Moyes & Miller, 2013). Moyes and Miller (2013) believe that the reason accurate empathy is so helpful is that vulnerable people often feel met with a degrading, judgmental and sarcastic approach. Thus, it is likely that the young people’s previous experiences of feeling treated like a case rather than a person made it especially significant that the mentors met them with authentic interest and respect.

### Strengths and limitations

A strength of the study is that the themes derived from the mentors’ interview were presented at the workshop with the mentors and further adjusted according to their comments. While the principle of respondent validation (Silverman, 2015) was thus followed with regard to the mentors, it would have enhanced the trustworthiness of the study, if the young adults’ had been given the opportunity to comment on the themes of the analysis as well. Another limitation of the study is that the first author carried out the preliminary text analysis alone (Smith, Flowers, & Larkin, 2009). However, the themes were discussed with the author group before the final interpretation and summarising. Furthermore, due to time constraints it was not possible to keep including participants until data saturation was reached. Thus, it is possible that the complexity of the participants’ experiences would have been described more thoroughly if more participants had been included in the study Saunders et al. (2018).

There are both advantages and disadvantages to conducting group interviews, rather than individual interviews. One of the advantages of interviewing the mentors together was that they appeared to be inspired by each other and expressed a desire to further develop their work and the young adult group. According to Halkier (2010a, 2010b)) this is one of the main advantages of interviewing several people together. On the other hand, there is a risk that participants in group interviews can become preoccupied with appearing in a positive light in front of the other participants, the so-called *social desirability bias*, which may result in less honest answers (Richman, Weisband, Kiesler, & Drasgow, 1999). This could especially have been the case in the mentors’ interview, where the manager was part of the group, thereby creating an unequal basis of power, where the mentors could have been reluctant to share some of the things they experienced as being difficult in the group to appear in a positive light. While this may definitely be a limitation, the interviewer attempted to provide the best conditions for honesty and to strengthen the relationships between the participants by asking questions about the participants’ intentions and values. Furthermore, it was the impression of the interviewer that the manager and the mentors had a good relationship because the manager had been working closely together with the mentors previously and was recommended for the position as manager by the mentors.

## Conclusion

The young adults in the RENEW group highlighted the mentors being authentic and personally engaged in the young people as the most helpful aspect of the group. The authenticity can also be observed in the way the young people experienced many of the activities in the group as spontaneous input from the mentors rather than prescribed exercises. The young adults furthermore described helping each other and doing things together as something important. The function of the group was challenged by the compulsory attendance requirement and the pressure on the mentors’ time. The structural, institutional, conditions appear to have had negative consequences for the mentors’ work with the group and the relationship between the young adults and the mentors, and thus potentially for the young adults’ rehabilitation process as well. In future interventions for vulnerable young adults it is important that adequate resources are made available for team cooperation around the young people and it may also be necessary to relax, or to change, counterproductive formal rules.

The study shows that meeting young adults authentically and flexibly with some self-disclosure is experienced as helpful—also in manual-based group interventions such as RENEW. That the intervention was delivered spontaneously must be attributed to the personal commitment of the mentors, to the fact that the original manual was created in collaboration with the mentors, and to fact that the underlying values are in agreement with those of the mentors.
